# Significant improvement of laparoscopic knotting time in medical students through manual training with potential cost savings in laparoscopy - an observational study

**DOI:** 10.4274/jtgga.galenos.2020.2020.0019

**Published:** 2020-09-03

**Authors:** Sebastian Findeklee, Georg-Peter Breitbach, Julia Caroline Radosa, Emanuela Morinello, Elmar Spüntrup, Erich-Franz Solomayer, Carolin Spüntrup

**Affiliations:** 1 Clinic for Gynaecology, Obstetrics and Reproductive Medicine, Saarland University Hospital, Homburg, Germany; 2 MVZ Fertility Center Hamburg, Hamburg, Germany; 3 Clinic for Anaesthesiology, Intensive Care and Pain Management, Saarland University Hospital, Homburg, Germany; 4 Clinic for Radiology, Saarbrücken Hospital, Saarbrücken, Germany; 5 Pelvic School Saarbrücken, Saarbrücken, Germany

**Keywords:** Laparoscopy, laparoscopic knotting, surgery simulator, equivalent calculation

## Abstract

**Objective:**

Laparoscopy is a standard procedure in operative gynaecology, but laparoscopic simulator training for novices/junior surgeons is not currently well-established. The aims of this study were to demonstrate that a laparoscopic knot course for trainees can significantly shorten the knotting time and to perform a counter-value calculation for the clinic’s costs.

**Material and Methods:**

An observational study was performed with exercises on a laparoscopic box trainer as part of the practical clerkship in gynaecology and obstetrics between 07.10.2019-31.01.2020. At the beginning and at the end of the exercises, the participants made a laparoscopic knot and the difference in knotting time, Δt in seconds (s) was measured.

**Results:**

Eighty-eight medical students needed an average of 247.1 s for the first laparoscopic knot at the beginning of the course and an average of 45.43 s for the second at the end of the course. Mean shortening of the knotting time was 201.67 s or 81.6% (p=0.02). Calculating costs of an average of €40-50 for an operation minute would mean a cost saving of at least €120-150 for a partial node.

**Conclusion:**

Trainees can significantly improve their operative skills in a short time with the aid of surgical simulation training. Such training can be beneficial for clinics by reducing the operating time if the basics, such as sewing and instrument guidance, are learned on a simulator. We therefore suggest that operative simulation training should be mandatory in medical education.

## Introduction

Minimally invasive endoscopic surgery is now considered a standard procedure in many surgical fields, especially in gynaecology and urology, because it is associated with shorter convalescence and an improved cosmetic result. At the same time, it is characterized by a low peri- and post-operative complication rate (1). However, this depends less on the technical equipment in the operating room than on the training status of the surgeon (2).

According to Gallagher and Satava (3), laparoscopic surgeons can be divided into novices, juniors and experts with regard to their training status. Novices have performed less than 10 operations, juniors 10-100 and experts performed more than 100 operations (3). Laparoscopic surgery is sometimes characterized by relatively flat learning curves. Somewhat complex surgical steps, such as the laparoscopic knot should therefore not primarily be learned and practiced on humans. On the other hand, it seems to be essential to familiarize young doctors and possibly also medical students with the laparoscopic approach and to allow them to practise laparoscopic steps in order to be able to generate enough experienced young surgeons for the future. In order to reach this goal, various surgical simulators have been developed over the past few years. With the help of simulators, surgical skills can be practiced without endangering the patient (4). Unfortunately, training on surgical simulators has so far not been part of student teaching or medical training in operative subjects. Therefore, surgical training on simulators has so far been sporadic and not standardized (5).

**Figure 1:**
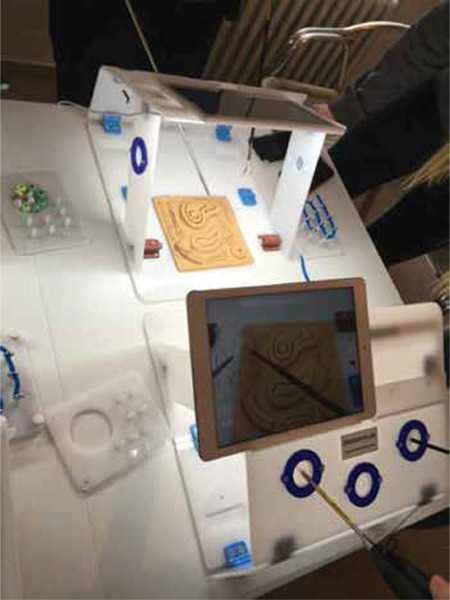
Structure of the laparoscopy simulator

The aim of this study was to measure the influence of manual training on the knotting time of medical students, as part of the practical clerkship in gynaecology and obstetrics. A classic box trainer working with a tablet camera was used and manual exercises were carried out in a defined sequence. Figure 1 shows the box trainer.

## Material and Methods

This was an observational study involving medical students as part of the practical clerkship in gynaecology and obstetrics in the winter semester 2019/2020. The study period was from 07.10.2019 to 31.01.2020.

The training protocol was as follows. There was one tutor (study doctor with experience in laparoscopic surgery) for a group of six to eight students. At the beginning of the course, the course participants were asked to perform a laparoscopic knot with a wrapping on a self-developed, laparoscopy simulator, including a box trainer and a tablet camera. This wrapping and the functionality of the instruments were explained to the students in advance. After the students had briefly familiarized themselves with the instruments, they started with their knot. The knotting time in seconds (s) was measured by the study doctor. For this first part of the training, including instruction by the doctor and knotting, a total of 30 minutes were allowed.

The following second training step lasted 30 minutes. Here, the students carried out hand-eye coordination exercises with one and two arms and skill training according to a defined protocol. This protocol included picking up tacks with a pair of pliers in the box trainer, threading beads onto a stick and running a ring over a splint without touching it using a laparoscopic needle holder.

Subsequently, clamping a needle in the correct way was demonstrated and the looping for the knot was repeated. The students were now asked to carry out different kinds of seams (interrupted suture, continuous rows of seams). The seams were secured with a double-strand knot and a counter-rotating single knot. This third training session also took 30 minutes. The respective seams and knots were made with braided and thin monofilament threads, so that the students could also develop a feeling for different thread sizes and thread types.

Finally, in the last 30 minutes of the knotting training, the students were asked to perform a laparoscopic knot on the simulator again. The study doctor measured the knot time in seconds for a second time. The difference between the mean knotting time of the first and last laparoscopic knot (∆t) was measured. At the end, every student gained feed-back from study doctor.

**Figure 2:**
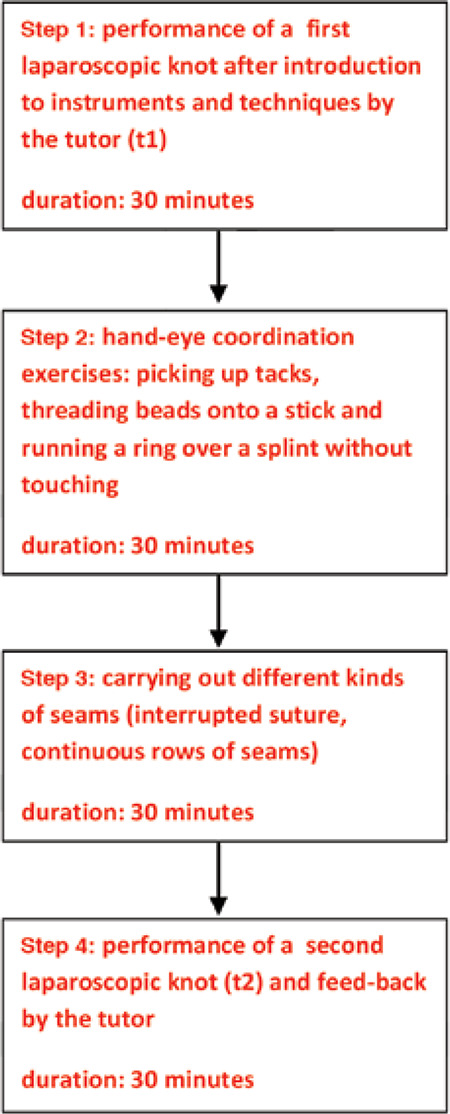
Course of the laparoscopic knotting training

The training with the four modules, each 30 minutes long, lasted a total of two hours. The course of the laparoscopic knotting training is summarized in Figure 2.

### Statistical analysis

The paired t-test was used to check whether there was a significant difference between the mean knotting time of the first endoscopic node (t1) and the mean knotting time of the second laparoscopic node (t2) (significance level p<0.05). Statistical analysis was performed with the aid of SPSS version 24 (IBM, Armonk, New York, USA).

### Informed consent and ethics

All study participants signed an informed consent form for further processing the obtained data anonymously before participating in the training course. The local institutional review board was contacted to ask for an ethics vote for the study, but this was not required for the study since it was a regular course belonging to the practical clerkship in gynaecology and obstetrics within the student curriculum.

## Results

Between 07.10.2019 and 31.01.2020, a total of 88 medical students took part in the laparoscopic node course with the laparoscopy simulator. The mean time to complete the first laparoscopic knot at the beginning of the course was 247.1 s (minimum: 45 s, maximum: 1,290 s, range: 1,245 s, median: 790 s). An average of 45.43 s (minimum: 7 s, maximum: 280 s, range: 273 s, median: 150 s) was required for the second laparoscopic knot at the end of the course. Thus the knotting time was shortened by 201.67 s or 81.6% due to the learning success with the help of the course (Table 1). The difference between the mean first knotting time and the mean second knotting time (∆t) was statistically significant in the paired t-test (p=0.02). Calculating costs of an average of €40-50 for an operation minute would mean a cost saving of €120-150.

## Discussion

Our study shows that medical students can significantly reduce their knotting time by an average of 81.6% after attending a laparoscopic knotting course using a laparoscopy simulator, performing skill exercises and with adequate demonstration and explanation of the knotting technique. In this specific case, the clinic could save €120-150 for each laparoscopic partial node performed if one node had calculated costs of €40-50 per minute of surgery time, which seems realistic with regard to the published literature (6).

Whereas laparoscopic surgery in general possesses a low operative complication rate, it is self-evident that expertise increases with the surgeon’s experience. From an ethical point of view, it should not be a requirement for scientific evidence of an economic benefit for the clinic before implementing laparoscopic simulation courses into specialist training for medical doctors, improved clinical performance and safety should be sufficient. In our opinion, it does not appear ethically responsible to carry out complex surgical interventions in humans with the risk of serious complications without having practiced the individual surgical steps beforehand. Even if there are no complications intra-operatively, such as organ injury, the longer duration of the operation with longer anaesthesia can pose a risk to the patient, especially in the case of pre-existing diseases. There is evidence that complication rates are directly related to surgical duration in gynaecological surgery (7). As laparoscopic simulator training enables a significant shortening of surgical partial steps like knotting it also has the potential of reducing operative complication rates.

Since longer anaesthesia also means an increase in costs for the clinic, these examples stress how closely linked the medical and economic consequences of longer operation times are. In the same context it has to be stressed that ethical and economic aspects are not mutually exclusive. Resources in every health care system are limited - hence a responsible use of health care resources, such as operating time, is important so that limited health care resources can be available to as many patients as possible.

Comparable to the pilot training that has been established for decades, there are currently numerous simulators for laparoscopic operations available with which comparable successes have been demonstrated with respect to learning curves (8,9,10). However, training on the simulator in the surgical curriculum - in contrast to pilot training - is not intended for surgeons. In contrast to pilots, the costs must also be borne by the trainee her- or himself. Hospital providers often argue that they cannot cover the costs of surgical skill training on the simulator, because a counter value calculation cannot be directly derived. In addition, up to now there is insufficient data on whether both surgery time, as the most cost-intensive factor, and the operative complication rate can be reduced by surgeons trained on the simulator, so that ultimately patients, hospital operators and young surgeons could benefit from the simulated training (3,11).

Our study provides new data on this issue. It was possible to measure a significant reduction in knotting time through structured simulator-based training. This could result in potential savings in the three-digit Euro range for each laparoscopic partial node. A countervalue calculation also has to consider the exact costs of simulation training. At the same time, it must be emphasized that the costs of structured laparoscopic knotting training on the operation simulator are very low. The technical equipment with box trainer and tablet camera amounts to less than €1,500 (box trainer €280, tablet camera approximately €200, laparoscopic instruments including needle holder, grasping forceps and scissors approximately €1,000). The costs of the suture material are about €250 per package including 36 pieces. Suture material and endoscopic instruments are also available in every clinic. It is difficult to calculate the costs for the tutor because the study doctor is employed at the clinic and can take the course during working time. There was no necessity for an additional salary. The training programme for all 88 participants at our institution incurred costs of approximately €2,250. Fortunately, the technical equipment is re-usable for future trainings. Calculating the operating costs at €50 per minute, this would mean that the simulator training would have paid for itself if cumulative knotting time of all 88 participants together could be reduced by at least 45 minutes. The effect of our training was a total reduction in knotting time of about 296 minutes. We therefore recommend our training concept, based on our experience, as it is clinically beneficial for the trainees and is also financially worthwhile. Our training concept also allows a variety of other exercises relevant to the operating room, such as of spatial imagination within the laparoscopic site or hand-eye coordination.

A decrease in the rate of operative complications after training with the simulator cannot be directly derived from our study. However, if we regard the handling of the instruments by the students before and after the course, it does not seem unconscionable to expect a potential for reduced surgical complications due to an increase in confidence utilizing laparoscopy instruments. For example, after the training, the seam pad, possessing a toughness comparable to that of intestinal tissue, showed significantly fewer tears of the monofilament threads. Perhaps, this could result in a lower rate of anastomotic leakage in the case of surgical interventions on the intestine.

There are several other studies demonstrating that laparoscopic techniques like knotting or sutures can also be learned by novices with the aid of simulation courses (12,13,14,15). In contrast to previous studies, we have introduced laparoscopic simulation training into the gynaecological practical clerkship which offered the opportunity to observe a significant number of participants and to make a comparison of the time saving of laparoscopic knotting time with a potential saving in operation costs. Our study results therefore provide new evidence for young surgeons as well as medical faculties and teaching hospitals to make laparoscopic simulation courses an obligatory part of medical training or to have the course costs reimbursed by the employer.

Although surgical simulators are not a new invention, they are still not very widespread, because of the reasons given above. Basically, animal models as well as self-made plastic simulators without material from living beings can be used for practicing surgical interventions (16). From an ethical point of view, simulators made of non-biological material should be preferred. In addition, they offer the opportunity to practice surgical steps repetitively without time or space restrictions.

Laparoscopy simulators have been successfully evaluated in the past. Among other things, it could be shown that repetitive training has a greater influence on the success of learning the endoscopic knot than talent factors such as manual work or the desire to work in a surgical subject in the future (17). In addition, Ghesquière et al. (18) and Madec et al. (19) showed that surgical simulators are a suitable methodology for teaching surgeons appropriate laparoscopic technique. However, disadvantages of these studies are the relatively small number of participants and their monocentric character.

### Study Limitations

Our observational study also has some limitations. With 88 students, the number of participants is limited and does comprise just one centre. The examined laparoscopic node represents only a partial step of a minimally invasive surgical intervention. It is not possible to infer the improvement in the time required for the entire operative procedure. Besides, there was no further analysis of the characteristics of the students. For example, it could be that a disproportionately large number of students have already worked in a surgical field and therefore had easier access to the laparoscopic node. However, the “talent factor” argument was already invalidated by our publication from 2019 (17). It should also be considered that the number of participants in our study is distinctly higher than the previous publications that have dealt with this topic. Additionally, we are aware that the long-term retention of technical skills acquired during simulation training is a problem. For improving retention of skills, further regular training sessions are recommended and necessary. This circumstance also has to be factored in the cost analysis. Fortunately, repetitive training at an existing simulator is possible without obstacles and does not provide additional costs. Just the costs for the suture of about €250 per package including 36 pieces have to be estimated. A pragmatic solution could be seen in the utilization of expired sutures.

Furthermore, it has to be taken into account that different health care systems in different countries may go along with different health care costs including the costs for operating time. For example, a literature review performed by Chen et al. (20) revealed differences by a factor of two in operating costs in the different regions of the world (operating room costs per minute differing from $13.90 in Europe, the Middle East and Africa to $24.83 in North and South America) (20). Perhaps, this could lower the economic efficacy of simulation training in other countries independent from the ethical point of view.

In our opinion, the special finding of our observational study is that it is not just demonstrating a significant shortening of the time for a laparoscopic knot performed by inexperienced surgeons, but also provides a countervalue calculation for the potential saving of operation costs. If one assumes that a total laparoscopic hysterectomy requires at least two laparoscopic knots, this operation alone could save the clinic €250-300 with the aid of surgical skill training. For urogynaecological interventions with a mesh insert, this saving could be increased to €700-1,000. This could create an argument for the assumption of costs for operative simulation training by hospital authorities, from which doctors and patients would ultimately benefit. The fact that students regularly do not perform laparoscopic knots in real life may limit the impact of our findings. Nevertheless, the students from today are the doctors of tomorrow and also students during practical clerkship assist in the operating room with the opportunity of benefiting from acquired practical skills.

The lesson we learned from our laparoscopic training course is that it will be a mandatory component of the practical clerkship in the subject gynaecology and obstetrics at our faculty. Additionally, the simulator is currently used by young residents in our clinic. It is imperative to carry out practical exercises on the simulator under supervision before an operation can be performed in the operating room. Furthermore, the surgical and the urological clinic at our university are planning to implement a similar surgical skills training based on the positive experience we have gained with our course.

Finally, our study could help to further support the spread of operative simulation training, both in the context of training young surgeons and in the curriculum for teaching medical students at the universities.

## Conclusion

Young surgeons can significantly improve their operative skills in a short time with the aid of surgical simulation training. Such training on the simulator can be beneficial for the clinics by reducing the operating time if the basics such as sewing and instrument guidance are learned on the simulator. We therefore suggest that operative simulation training should be widely implemented in medical education.

## Figures and Tables

**Table 1:**
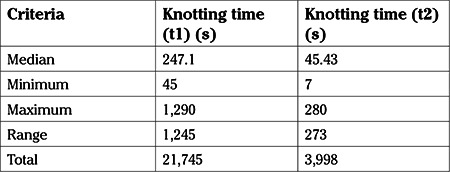
Comparison of knot times before and after the course
